# Traumatic myiasis in free-ranging eland, reported from Kenya

**DOI:** 10.1186/1756-3305-6-89

**Published:** 2013-04-08

**Authors:** Vincent Obanda, Ephantus Muthike Ndambiri, Edward Kingori, Francis Gakuya, Olivia Wesula Lwande, Samer Alasaad

**Affiliations:** 1 Veterinary Services Department, Kenya Wildlife Service, P.O. Box 40241–00100, Nairobi, Kenya; 2 Department of Medical Laboratory Science, Mount Kenya University, P.O. Box 342–01000, Thika, Kenya; 3 Estación Biológica de Doñana, Consejo Superior de Investigaciones Científicas (CSIC), Sevilla, Spain; 4 Institute of Evolutionary Biology and Environmental Studies (IEU), University of Zürich, Zürich, Switzerland

**Keywords:** *Taurotragus oryx*, *Chrysomya bezziana*, *Lucilia cuprina*, *Lucilia sericata*, *Myiasis*, Disease outbreak, Treatment

## Abstract

**Background:**

For centuries, immature stages of Dipterans have infested humans and animals, resulting in a pathological condition referred to as myiasis. Myiases are globally distributed but they remain neglected diseases in spite of the great medical and veterinary importance. Moreover, there is a paucity of information on the clinical-pathology and/or epidemiology of the infestation, especially in African free ranging wildlife.

**Findings:**

In the present study we report for the first time an outbreak of traumatic cutaneous myiasis (caused by Old World screwworm, *Chrysomyia bezziana* and blowfly, *Lucilia sp.*) in free-ranging common elands (*Taurotragus oryx*). The infestation affected both animal sexes and different age classes, and had a negative impact on individual fitness as well as the overall health. Severely affected individuals were euthanized, while others were clinically treated, and apparently recovered.

**Conclusions:**

This study indicates that myiasis-causing flies still exist in Kenya and are able to cause severe outbreaks of clinical cutaneous myiasis in wild animals. The status of these parasites in Kenya, which are of zoonotic potential, are either unknown or neglected.

## Findings

Arthropods such as mosquitoes, sandflies, ticks and dipteran flies among others are universal vectors of numerous pathogens that cause severe disease in humans and animals. For centuries, immature stages of Dipterans have infested humans and animals which, for a period, feed on the host's dead or living tissue, liquid body substances, or ingested food, resulting in a pathological condition referred to as myiasis [1]. The infestation occurs when an injury on a vertebrate host and ensuing foul smell attracts gravid female flies to oviposit on the edge of the wound and the hatched larvae burrow deep into the necrotic and sometimes living tissues of the host. Myiases are globally distributed, caused by diverse species of two-winged flies and have existed for centuries, but it remains a neglected disease in spite of the heavy economic losses to the livestock industry [2]. Myiasis–causing flies are not only agents of pathology but have been beneficially used medically in debridement therapy [3,4] and forensics to indicate time of death in homicide and wildlife poaching cases [5,6]. There are two approaches used to classify forms of myiasis; entomologically (obligate, facultative, accidental/pseudomyiases) [1,7] or clinically (when based on affected part of the host body). For instance, nasopharyngeal myiasis involves either aural or ocular myiasis or both especially when larvae infest several head cavities such as sinuses, mouth and ears [8].

New World screwworm, *Cochliomyia hominivorax,* Old World screwworm, *Chrysomya bezziana*, blowflies, *Lucilia sericata* and *L. cuprina* (Calliphoridae) are some of the common flies that cause traumatic/wound myiases of great medical and veterinary importance [9,10]. Although myiasis is known to occur in wild animals [1,11], not much information is available to describe the clinico-pathology or epidemiology of the infestation [12] especially in African free ranging wildlife [13].

In the present study we report for the first time to our knowledge an outbreak of traumatic myiasis in free-ranging common elands (*Taurotragus oryx*). The infestation had a negative impact on individual fitness and health. Severely affected individuals had to be euthanized, while others were clinically treated.

## Kigio Wildlife Conservancy (KWC)

The study area was Kigio Wildlife Conservancy (KWC), which is at 37M 0214547 UTM9920743, located between Naivasha and Gilgil towns, about 100 km west of Nairobi, in Kenya. The conservancy that is ring-fenced occupies 3500 acres. The fencing aims to inhibit entry of people and livestock and also demarcate the sanctuary. KWC is home to large populations of diverse wildlife species such as the endangered Rosthschild Giraffe (*Giraffa camelopardalis rothschildi*), common Zebra (*Equus quagga*), African buffalo (*Syncerus caffer*), Olive baboons (*Papio anubis*), Warthogs (*Phacochoerus africanus*), and medium-sized antelopes such as Waterbuck (*Kobus ellipsiprymnus defassa*), Impala (*Aepyceros melampus*), Thomson’s gazelle (*Gazella thomsonii*). The conservancy holds 180 common elands. KWC is neighboured with other farms such as Marula, which practices mixed-game ranching that includes cattle and wild ungulates, though there were no reported cases of myiasis outside of KWC.

### Ethics

The Committee of the Department of Veterinary and Capture Services of the Kenya Wildlife Service (KWS) approved the study including animal euthanizing capturing and treatment protocols. KWS guidelines on Wildlife Veterinary Practice-2006 were followed. All KWS veterinarians were guided by the Veterinary Surgeons Act Cap 366 Laws of Kenya that regulates veterinary practices in Kenya.

## Results and discussion

In May 2012, elands at Kigio Wildlife Conservancy (KWC) developed bleeding lesions on the ears, eyes and head that resulted in ill-health and necessitated clinical investigation and treatment. Between May and December 2012, a total of 10 elands (8 females and two males) were sick and 3 of them, which were the most severely affected, were euthanized. Out of the 10 elands, 8 were adults and two sub-adults (between 2.5 and 3.5 years old). The sick elands were observed to be listless, tended to be isolated from the herds, frequently shook the head and vigorously flapped the ears that were most of the time abnormally drooped. The animals had staggering gait and apparently impaired vision as they bumped into trees/thickets. The sick elands were chemically immobilized at different periods for closer examination, sampling and treatment. The animals were darted using a combination of etorphine hydrochloride (M99®, Novartis, South Africa) and xylazine hydrochloride (Kyron, South Africa) [14]. After immobilization, whitish/creamy maggots were observed streaming out from the wounded parts, ears and nose. Ears were particularly stricken with lesions ranging from total erosion (absence); atrophy (reduced size) due to scar tissue formation, thickening and or folding of the pinna, and others mutilated half way resulting to a 'stump'. A peculiar, distinct, pungent odour permeated the infested tissue and the affected animal. The malodorous, reddish brown fluid, produced in the wounds drains and stains the hair around or below the wound. Post-mortem examination of the skin lesions on euthanized elands revealed that the maggots had burrowed deep inside the head causing necrotic cavities. In all animals, the inner part of the pinna was infested with ticks of the genus *Rhipicephalus*.

After sampling of larvae, the skin lesions were cleaned using water to remove pus and then the rest of the visible maggots physically removed. The skin lesions were then topically treated with hydrogen peroxide and rinsed with iodine. Broad spectrum antibiotic, Oxytetracycline dehydrate, (Alamycine LA 300, Norbrook Laboratories Ltd, Northern Ireland) was injected (30mgs/kg body weight) intramuscularly. To control tick infestation, we applied Cypermethrin (Ectopor®, SA020, Novartis South Africa (Pty) Ltd) topically on the animals, and we administered Ivermectin (E-mectin ONE®, Eagle Vet. Tech Co. Ltd, Korea) subcutaneously at a dose of 1 mg/kg body weight. Four of the treated individuals were tagged to aid post-treatment monitoring. It took about 3 weeks post-treatment for these treated and tagged elands to recover and re-join herd groups.

The maggot samples were collected deep into the wounds and preserved in 10% buffered formalin and transported to the Forensic and Genetics Laboratory at Kenya Wildlife Service in Nairobi for identification. Diagnostic features of the larva such as the shape, size, spines, form of spiracles and cephalopharyngeal skeleton were examined using the identification key by Spradbery [9]. The larvae were dissected and cleared by lactophenol and then examined using a light microscope and micrographs were processed by Leica application suite, LAS EZ v 2.0.0 (Leica Microsystem Ltd, Switzerland).

**Figure 1:**
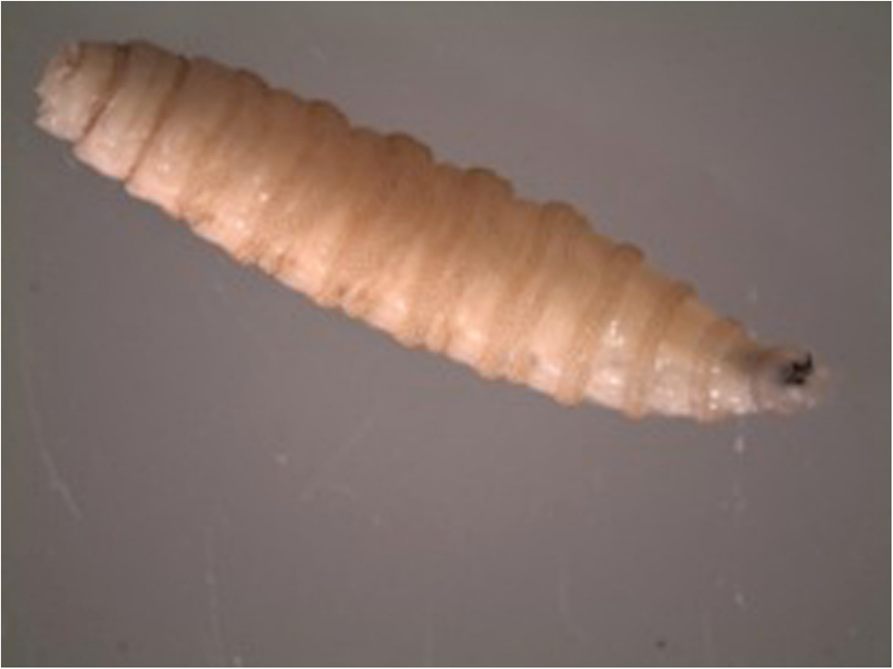
**Third stage larva of Old World screwworm, *Chrysomyia bezziana *(Calliphoridae), (Scale: 2000 μm).**

**Figure 2:**
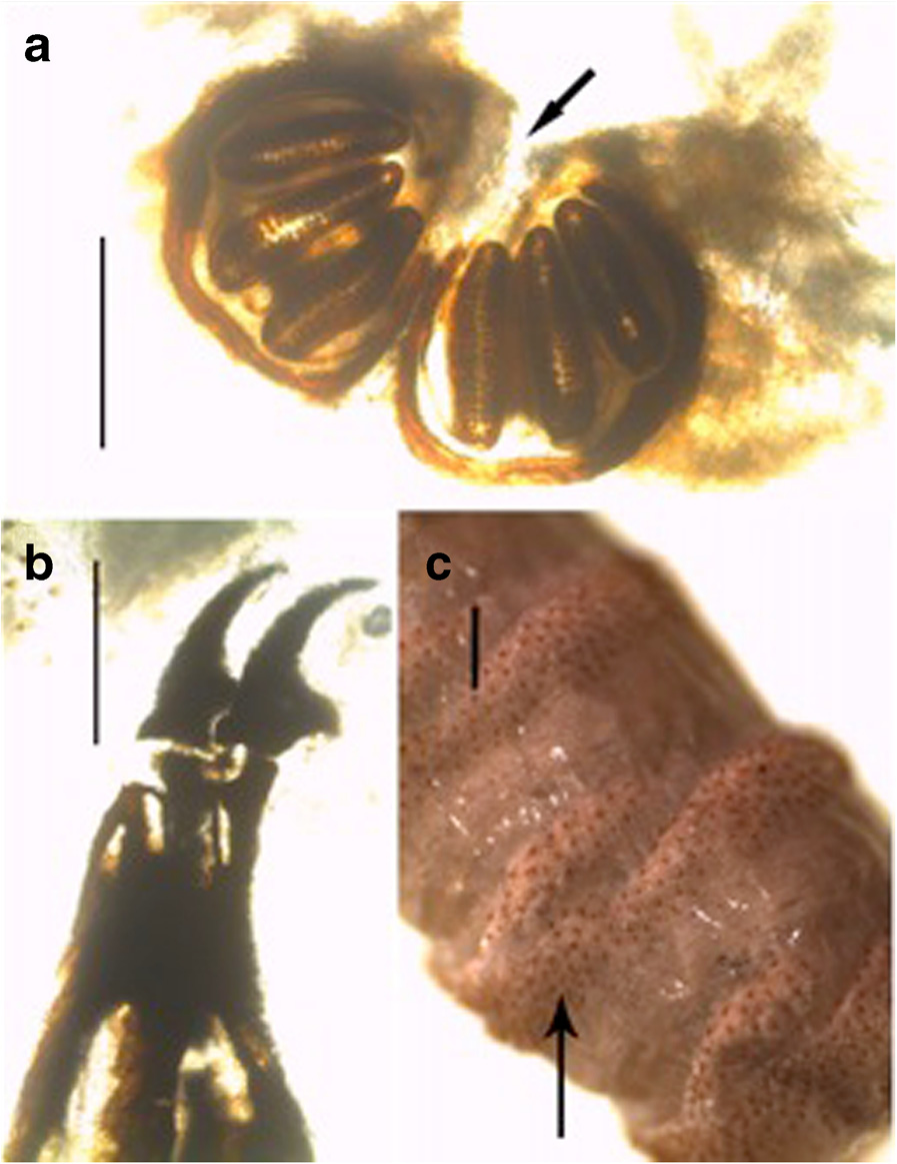
**(a) Posterior spiracle of third-stage larva of *Chrysomyia bezziana *(scale: 200 μm); the peritreme is incomplete (open) and button is indistinct (black arrow). (b)** Cephalopharyngeal skeleton attached to mouth-hooks that lack sclerite (scale: 200 μm). **(c)** Bands of somatic part spines of third-stage larva (black arrow), (scale: 500 μm).

**Figure 3:**
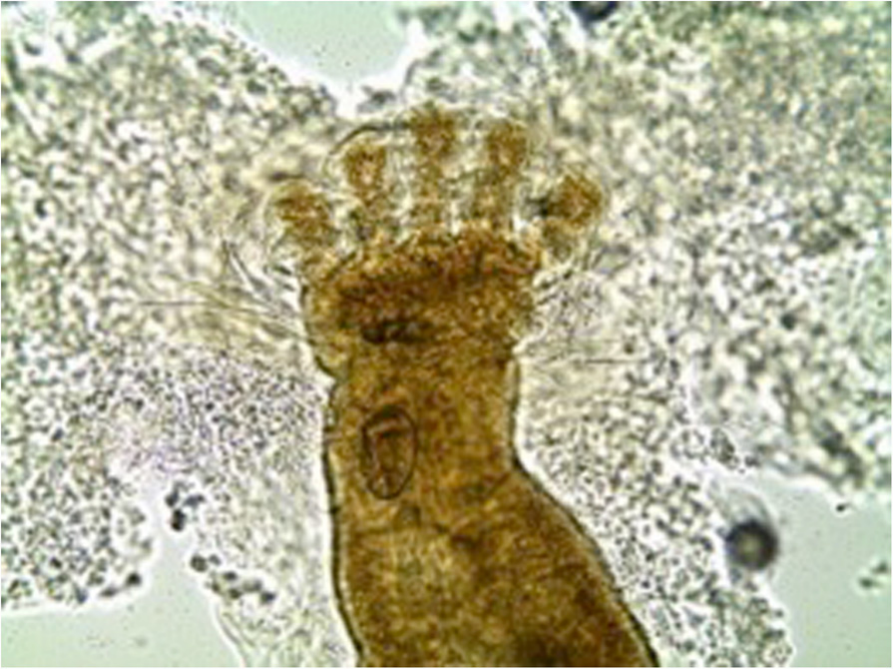
**The anterior spiracles of third stage larva of *Chysomyia bezziana *(Calliphoridae) with four lobes ×400.**

The third-stage larvae (n = 13) were microscopically examined and two different species identified. The common species (n = 12) was identified as *Chrysomya bezziana* based on the following features: the size of the larvae ranged from 10-12 mm and were not hairy but had prominent rings of spines (Figure 1); the posterior spiracle was visible, the pair of peritreme was incomplete (open), button indistinct and slits of the spiracles were straight (Figure 2a). Cephalopharyngeal skeleton attached to mouth-hooks that lack sclerite (Figure 2b). Cuticular spines were curved backwards to the posterior end of the larva (Figure 2c). Anterior spiracles had four lobes (Figure 3).

**Figure 4:**
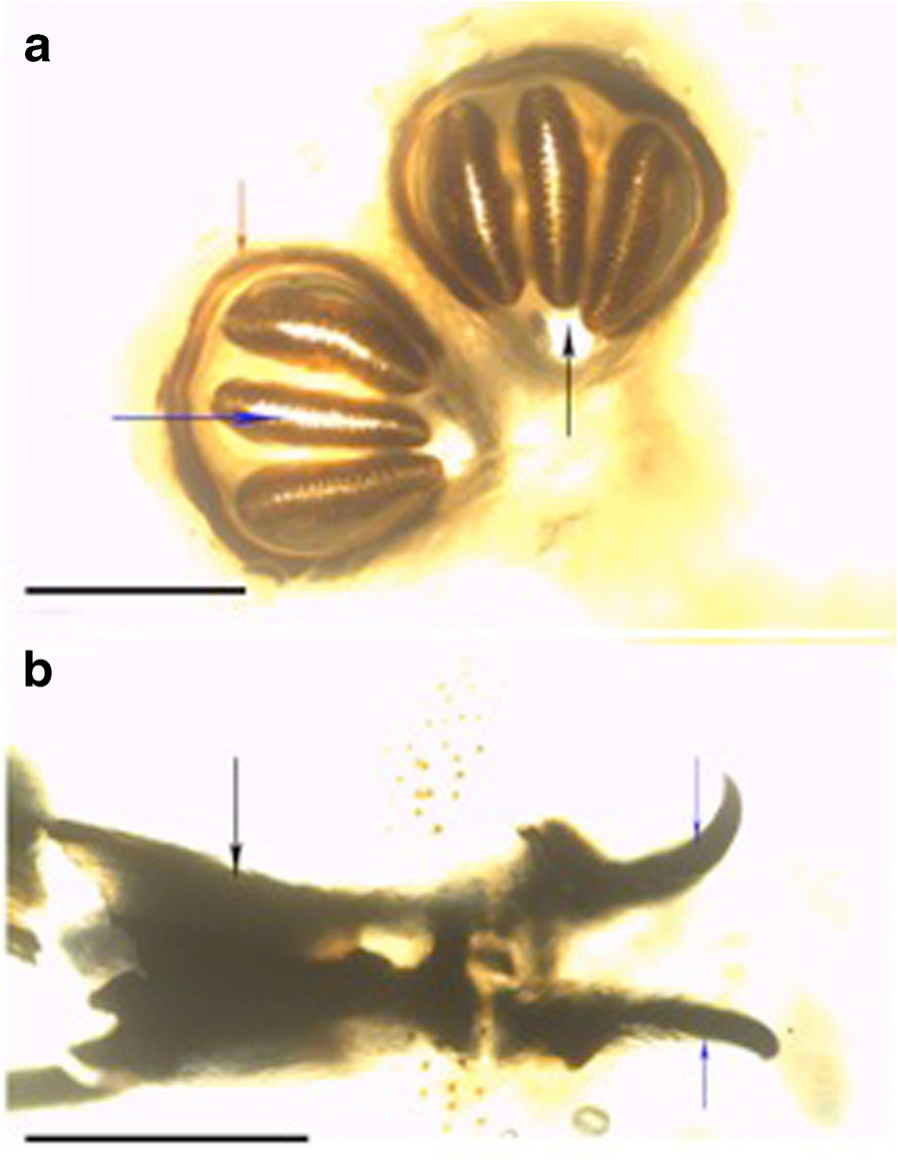
**(a): Posterior spiracles of third stage-larva of *Lucilia *spp. red arrow showing enclosed peritreme with thickened wall: blue arrow showing spiracle slits and back arrow showing enclosed button (scale: 200 μm). (b)**: Cephalopharyngeal skeleton (black arrow) attached to mouth-hooks (blue arrows) that lacked accessory oral sclerite (scale: 200 μm).

A single larva was identified to be *Lucilia sp.* based on the following features: The posterior spiracles were round with thick peritreme that enclosed a distinct button (Figure 4a). There was no accessory oral sclerite inbetween mouth-hooks (Figure 4b).

**Figure 5:**
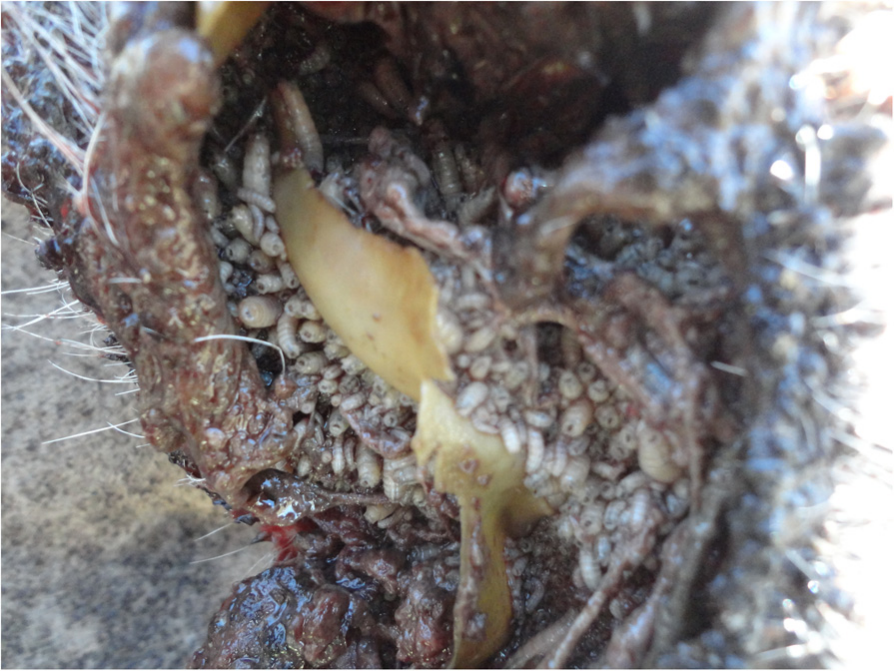
**Photo showing larvae (*in situ*) affecting the ear of one of the studied common eland.**

The present case is the first record of an outbreak of wound myiasis in common elands (Bovidae) in Kenya. The myiasis, which was caused by mixed infestation of Old World screwworm, *Chrysomyia bezziana* and eventually blowfly, *Lucilia sp.,* was clinically cutaneous due to extensive wounds on the ears, eyes and head. The infestation resulted in emaciation, malaise and some of the wounds were so severe that euthanasia was recommended. It seems that infestation reduces animal fitness as affected animals were emaciated and apparently lost hearing and balance, a state that makes them more vulnerable to predation. This may explain the scarcity of published literature on the incidence or epidemiology of cutaneous myiases in free ranging wildlife, besides possible challenges of tracking individuals having myiasis in the wild. The existing literature on myiasis in wildlife especially in Africa is old and scanty [1,11,13], which may fallaciously imply that the causal organisms are no longer present or infestations do not occur. The parasites identified in the present case concur with early records of myiasis-causing flies in Kenya: *L. sericata, C. bezziana, C. marginalis, Cordylobia anthropophagi, Sarcophaga haemorrhoidalis, Auchmeromyia luteola*[15]. Historical records of cases of myiasis in Kenya point out that infestation occurred on people and their livestock, with human cases ranging from minor to life threatening conditions. For instance, the larvae of *L. sericata* destroyed the cranium and exposed the brain of a woman that had nasopharyngeal myiasis [15]. Screwworms (Figures 1, 2 and 5) and blowflies (Figure 4) both belong to the family Calliphoridae but are morphologically distinct, yet common among the major groups of wound myiasis-causing flies that result in great production losses, morbidity and mortality in diverse species of livestock and wild animals [1,11,12]. In the genus *Lucilia*, which comprises of 27 species, *L. cuprina* and *L. sericata*, whose spatial occurrence overlaps in Africa, are the two species of clinical and economic importance as the primary cause of wound myiasis [9].

The present case involved mixed dipteran species whose larval feeding habits differ [9]. Screwworm larvae are known to be deep-feeders, burrowing deep into the wound while blowflies tend to feed superficially on the surface of the wound being nourished by the serous exudates from the host [9]. Upon disturbance, these larvae respond differently, with blowfly larvae having a rapid mass exodus from the wound while screwworms respond by retreating deeper into the wound [9]. This corresponds to the observation of larvae streaming from the wound when darted animals became recumbent, and further explain the few numbers of blowfly larvae sampled.

Large African mammals such as elephant, water-buffalo (*Bubalus bubalis*) [1], eland, black rhinoceros (*Diceros bicornis*), giraffe (*G. camelopardalis*), lion (*Panthera leo*), waterbuck, impala and bushbuck (*Tragelaphus scriptus*) [11] are recorded as susceptible to Myiases. One of the few published cases of clinical myiasis in wild animals involve *L. sericata* which caused severe gluteal traumatic myiasis in a gazelle (*Gazella subgutturosa*), that later died [12]. Records of clinical wound myiasis are rare in elands but Mlu *et al.*[16] observed infestation of elands with flesh fly *Wohlfahrtia sp.* (Sarcophagidae) in a Russian zoo. According to Mlu *et al.*[16], 24 different mammalian species were affected with myiasis in this zoo but infestation was more frequent in elands and American bison (*Bison bison)* with a prevalence of 33.5% and 9.4%, respectively. In the present case, infestation was more common in females compared to males and was not observed among juveniles. However, we could not draw statistical inference from these data because the sample size was small.

Among factors that may attract myiasis-causing flies to infest domestic animals are injuries of broken horns, injured eyes, lesions caused by ticks or ox-pecker (*Buphagus spp*.) and soiled fleece of sheep [15]. Some of these may have occurred in the studied elands. In the present case, myiasis was persistent on elands in spite of obvious sympatry with diverse mammalian hosts including other bovids. We suppose that since gravid flies use olfactory cues to locate wounds where they oviposit, [9], it is likely that once infestation had begun in elands, the resulting myiatic-wound odour oriented the flies [17] to these particular hosts, ignoring other potential host species in the habitat.

Probably more elands were infested in the population at KWC, however, the condition is only noticeable when the infestation is sufficiently severe to cause the animal to lose track of the herd and become isolated. Rejoining a herd after treatment is therefore suggestive that the individual has recovered and has regained fitness. Ivermectin and doramectin are among the common larvicidal agents [18,19], giving up to 100% efficacy in the treatment of myiasis in domesticated animals [20]. In the present case, we suggest that our treatment regime, which was based on Ivermectin, was effective on these wild elands.

This study indicates that myisias-causing flies still exist in Kenya and are able to cause a severe outbreak of clinical cutaneous myiasis in wild animals. The status of these parasites in Kenya, which are of zoonotic potential, is either unknown or neglected.

## Competing interests

The authors declared that they have no competing interests.

## Authors’ contributions

VO, EMN, EK and OWL conceived and designed the experiments. VO, EMN, EK, FG, OWL and SA performed the fieldwork experiments. Manuscript was analysed, discussed and written by all co-authors. All authors read and approved the final version of the manuscript.
